# Can we learn from hidden mistakes? Self-fulfilling prophecy and responsible neuroprognostic innovation

**DOI:** 10.1136/medethics-2020-106636

**Published:** 2021-07-12

**Authors:** Mayli Mertens, Owen C. King, Michel J. A. M. van Putten, Marianne Boenink

**Affiliations:** 1 Center for Medical Science and Technology Studies, Department of Public Health, University of Copenhagen, Copenhagen, Denmark; 2 Department of Philosophy, University of Twente, Enschede, Overijssel, The Netherlands; 3 MIRA-Institute for Biomedical Technology and Technical Medicine, University of Twente, Enschede, Overijssel, The Netherlands; 4 Department of Clinical Neurophysiology, Medisch Spectrum Twente, Enschede, Overijssel, The Netherlands; 5 Department IQ Healthcare, RadboudUMC - Radboud University, Nijmegen, Gelderland, the Netherlands

**Keywords:** end-of-life, research ethics, neuroimaging, neuroethics, medical error

## Abstract

A self-fulfilling prophecy (SFP) in neuroprognostication occurs when a patient in coma is predicted to have a poor outcome, and life-sustaining treatment is withdrawn on the basis of that prediction, thus directly bringing about a poor outcome (viz. death) for that patient. In contrast to the predominant emphasis in the bioethics literature, we look beyond the moral issues raised by the possibility that an erroneous prediction might lead to the death of a patient who otherwise would have lived. Instead, we focus on the problematic epistemic consequences of neuroprognostic SFPs in settings where research and practice intersect. When this sort of SFP occurs, the problem is that physicians and researchers are never in a position to notice whether their original prognosis was correct or incorrect, since the patient dies anyway. Thus, SFPs keep us from discerning false positives from true positives, inhibiting proper assessment of novel prognostic tests. This epistemic problem of SFPs thus impedes learning, but ethical obligations of patient care make it difficult to avoid SFPs. We then show how the impediment to catching false positive indicators of poor outcome distorts research on novel techniques for neuroprognostication, allowing biases to persist in prognostic tests. We finally highlight a particular risk that a precautionary bias towards early withdrawal of life-sustaining treatment may be amplified. We conclude with guidelines about how researchers can mitigate the epistemic problems of SFPs, to achieve more responsible innovation of neuroprognostication for patients in coma.

## Introduction: self-fulfilling prognosis for coma patients and the clinical dilemma

Consider Hans who at 62 has a sudden cardiac arrest. His blood circulation stops and, as a result, his brain is deprived of oxygen and nutrients. Although Hans is successfully resuscitated, he remains unconscious and is brought to intensive care in a postanoxic coma where he receives life-sustaining treatment. Over the next few days, physicians use state-of-the-art prognostic techniques in an attempt to ascertain the extent of Hans’ brain damage. The available evidence suggests it is severe; the prognosis is poor. The treating physicians expect that if Hans were ever to regain consciousness at all, it would be with a prolonged disorder of consciousness and an extremely poor quality of life. After discussion, the physician and Hans’ family decide to withdraw life-sustaining treatment, allowing Hans to pass away peacefully. In light of Hans’ interests and those of his family, we take it that this can be a highly reasonable decision. However, how would we know if the prognosis was incorrect? If the prognosis was actually mistaken, we cannot learn from this mistake. This article explores the problems for responsible research and innovation in neuroprognostication that are raised by this sort of case, and suggests guidance for mitigating these problems.

The poor prognosis for Hans was a self-fulfilling prophecy (SFP), because it was a prediction that brought about its own fulfilment. In general, we define an SFP as a prediction that is employed, or acted on, in a way that affects the very situation that the prediction is about, and, due to this, the predicted outcome is realised.^1^ In saying that the outcome is due to the prediction and the way it is employed, we do not mean that these constitute the sole or entire explanation of the outcome. Rather, the prediction and the way it was employed play essential parts in the explanation of how the outcome was actually realised. For Hans, the poor prognosis and the decision to withdraw life-sustaining treatment was the proximal cause of his death, regardless of what his eventual fate might otherwise have been. Hence, the prognosis was an SFP.

In light of this, we argue that it is important to distinguish between two general classes of SFPs. In some cases of SFPs, the outcome realised (here, the death of the patient) would not have been actually realised without the prediction (here, prognosis of poor outcome) and the way it was employed (here, withdrawing life-sustaining treatment). In such cases, the SFP changed the outcome from what it otherwise would have been; this we call a *transformative SFP*. Although these cases are the most dramatic, we must immediately note that they are not the only cases of SFPs. In some other cases, the way a prediction is employed commandeers the process by which the relevant outcome is realised but without altering that outcome. In such a case, even if the outcome predicted would have been eventually realised in the absence of the prediction, it was the prediction and its employment that determined *how* it actually was realised. Since such an SFP does not change what the outcome would have been, it is not transformative; it is merely an *operative SFP*. An operative SFP takes over the process without changing the eventual outcome. In our analysis, we will show why it is essential to consider the implications of both transformative and operative SFPs.

Our analysis applies to SFPs across many practical and research contexts. In this paper, however, we focus on SFPs in neuroprognostication, especially for patients in coma after cardiac arrest.

Cardiac arrest, for nine out of ten patients, is immediately fatal.^3^ Yet, even successfully resuscitated patients usually do not immediately regain consciousness. They are taken to an intensive care unit in a state of postanoxic coma. During cardiac arrest, the brain is deprived of oxygen. Predicting the extent of the resulting neurological impairment and the patient’s eventual outcome is difficult. The range of outcomes is a spectrum between clearly good, as with full neurological recovery, and clearly poor. Clearly poor outcomes typically include vegetative state, other prolonged disorders of consciousness and death.^2^ In the hours and days after a patient is resuscitated but still comatose, the treating physicians (and the patient’s family, if they are involved in decision making) face a dilemma due to prognostic uncertainty: Either they withdraw life sustaining treatment and allow the patient to die peacefully, precluding the possibility of recovery, thereby realising an SFP. Or they continue treatment, in the hope of eventual recovery, with the risk that the patient regains consciousness without ever regaining an acceptable quality of life.^3^

Although similarly structured dilemmas occur with other dire judgements in intensive care, such as a diagnosis of brain death, the dilemma is especially pressing in cases of postanoxic coma. This is because of the high degree of uncertainty in the prognosis for these cases, combined with the fact that withdrawal of life-sustaining treatment (WLST) is the major cause of death for this patient group past the first 24 hours.^3^ ^4^ Hence, the dilemma in neuroprognosis for postanoxic coma is especially controversial.^7–9^

With so much uncertainty and such high stakes, the need to improve neuroprognostication is pressing. The goal of prognostic innovation is to improve certainty and thereby mitigate the severity of the dilemma we just introduced. Specifically, we want a high degree of certainty that (1) when the prognosis is good, continued treatment will indeed yield a good outcome and (2) when the prognosis is poor, continued treatment would not yield a good outcome.

Typically, clinicians, as well as most other stakeholders, focus on transformative SFPs.^10^ Because a transformative SFP brings about a death that would not have occurred otherwise, it gives rise to moral concerns.^7 8 11^ If the prognosis brings about a poor outcome (viz., death) when a poor outcome could have been avoided, then this is an unfortunate, tragic result. However, it is important to recognise that such cases, despite being tragic, are not necessarily morally objectionable. Prognosis, by its nature, takes place under conditions of uncertainty. Proceeding on the basis of the best evidence available is no guarantee of correctness, but it is the best we can do.

A moral problem arises only if the decision-makers fail to acknowledge that the poor prognosis and ensuing decisions ensured an outcome which had been hitherto uncertain. This would be morally problematic, as an avoidance of accountability.^12^ Although dealing with probabilities rather than certainties is inevitable, we expect medical professionals to acknowledge any role they play in resolving the uncertainty in the direction of one outcome or another and communicate this clearly to patients and their families.^10^ We mention this potential moral problem regarding SFPs in order to, first, acknowledge its importance, and, second, distinguish it from the further concern we wish to illuminate.

Our concern is fundamentally about the epistemology of SFPs in neuroprognostic research, to which we turn in the next section. In that section we also show why SFPs’ epistemic problems, though very pressing, are difficult to solve because of clinicians’ obligations to their patients (who are also the relevant research subjects). Having laid out the general epistemic problem with SFPs in neuroprognostication, we then proceed to detail three specific mechanisms by which this problem distorts research and innovation. This will put us in a position, just before we conclude the article, to note the risks of further moral problems due to the ways SFPs impede research.

## SFPs’ core challenge to responsibly innovating neuroprognostication

This section begins by articulating the core epistemic problem that SFPs pose for neuroprognostic research. Once we have clarified why this problem is pressing, we explain why—in light of clinicians’ duties to their patients—it is so difficult to avoid.

Prognosis is based on one or more tests measuring brain damage. A positive test result indicates a poor neurological outcome for the patient and encourages a decision to withdraw life-sustaining treatment. Of course, most test results are imperfect indicators; both true positives and false positives occur. The common concern about SFPs *in clinical practice* is the risk of harming some patients due to incorrect poor prognosis, bringing about a poor outcome that might have been otherwise averted. This is a moral problem due to unrecognised false positives, which yield transformative SFPs. The problem SFPs pose *in research*, however, is about the difficulty in distinguishing between false positives, yielding transformative SFPs, and true positives, which yield merely operative SFPs. In short, the problem in research is not so much a moral problem about the unnecessary, unfortunate patient outcomes, but rather an epistemic problem about the difficulty in telling the difference between the unnecessary unfortunate outcomes and ineluctable ones.

Clinicians and researchers have acknowledged that SFPs have both clinical and research consequences,^2 7 8 10–12^ but, so far, there has been inadequate recognition about the epistemic value (or lack thereof) of prognoses that were self-fulfilling. We can fully acknowledge the distinctive and dramatic significance of unrecognised false positives due to their immediate clinical consequences, while also correcting the oversight regarding how SFPs impact research. As we will now explain, when predictions bring about their own fulfilment, researchers can *never* rely on, as genuinely informative, any feedback they receive about the accuracy of those predictions. The added insight here is about exactly which instances of prognosis do—or do not—provide evidence relevant to the quality of the prognostic test.

To see the problem, return to the example of the fictional patient, Hans. Suppose that, according to a prognostic technique under development, Hans tests positive for severe brain damage. Ordinarily, running such a test would be an opportunity to assess the quality of that test, by comparing the outcome predicted on the basis of the test to the eventual actual outcome. In other words, we can assess the innovative test by checking whether the positive result was a true positive or a false positive. However, since life-sustaining treatment is withdrawn on the basis of the prediction, the prediction is an SFP, and Hans’s outcome is guaranteed to be poor. If the test was a true positive, it will appear as a true positive. But even if it was a false positive, it will appear as a true positive. There is no way of telling which is which. Hence, any apparent feedback we might seem to glean from observing the actual outcome of Hans’s case is dubious. Thus, the cases in which a test yields an SFP constitute an inherently unreliable source of feedback. Any inference about the quality of the test would be invalid (table 1).

**Table 1:** Feedback received when positive test results motivate withdrawal of treatment

POSITIVE TEST RESULTS Outcome predicted: poor	
**FALSE POSITIVE**	**TRUE POSITIVE**
Patient dies after life-sustaining treatment is withdrawn, based on the poor prognosis However, patient would have a good outcome (given continued life-sustaining treatment) Prognosis changes outcome **--> Transformative self-fulfilling prophecy**	Patient dies after life-sustaining treatment is withdrawn, based on the poor prognosis Regardless, patient would have a poor outcome (given continued life-sustaining treatment) Prognosis does *not* change outcome **--> Operative self-fulfilling prophecy**
**Outcomes observed: POOR (death of the patient**) **--> Unreliable feedback**	

In cases of self-fulfilling poor prognosis for a patient in coma, the explanation for the feedback’s unreliability is that, discriminating between false positives and true positives is practically impossible due to the death of the patient. Thus, except from exploring ways to confirm the prognosis postmortem,^13 14^ it becomes impossible to know whether the prognosis was transformative or merely operative.^5^

Compare this to the case of a negative test result, or any other test that does not yield an SFP. If the test result is negative, and the prognosis is good, then life-sustaining treatment will be continued.^6^ If the negative result was a true negative, we will observe a good outcome for the patient. In contrast, if the negative result was a false negative, we will observe a poor outcome for the patient. In the case of the false negative, we can catch our error. The observation of an outcome that was contrary to what we predicted constitutes an error signal (table 2). The problem with positive results yielding SFPs is that, with the death of the patient, they eliminate any possibility for an error signal to alert us that the test result was inaccurate. In contrast, negative test results (test results which do *not* yield SFPs) produce error signals in instances where the test was incorrect.

**Table 2:** Feedback received when negative test results motivate continuation of treatment

NEGATIVE TEST RESULTS Outcome predicted: good	
**FALSE NEGATIVE**	**TRUE NEGATIVE**
Life-sustaining treatment is continued based on good prognosis However, patient has poor outcome after continuation of treatment, based on the good prognosis (which is an error signal) Prognosis does *not* change outcome **--> No self-fulfilling prophecy**	Life-sustaining treatment is continued based on good prognosis Indeed, patient has good outcome after continuation of treatment, based on the good prognosis (yielding no error signal) Prognosis does *not* change outcome **--> No self-fulfilling prophecy**
**Outcome observed: POOR** **--> Reliable feedback**	**Outcome observed: GOOD** **--> Reliable feedback**

We have just seen that while we can learn from negative test results, it is impossible to learn from positive test results that produce SFPs. Medical practitioners are usually aware of the potential for transformative SFPs. However, the fact that, in cases of transformative SFPs, the outcome would have been different had the prediction not been made is a condition that pollutes the evidential value of all the similar predictions, including the SFPs that were merely operative. We cannot learn from a true positive if we cannot be certain it was not a false positive. Hence, when a patient’s case is also a basis of further research, clinicians and researchers must extend their awareness to *all* SFPs, including those that are merely operative. Failure to do so perpetuates a bias that produces false positives, and thus limits the value—and leads to misrepresentation of the value—of the innovations that are developed from this research.

The epistemic impediment caused by SFPs is of special importance, not for its immediate clinical consequences, but rather for the research and development of new prognostic tools and techniques. Innovation in prognostication for postanoxic coma is driven by the goal of reducing uncertainty that causes clinical dilemmas regarding coma patients. As we have emphasised from the beginning, desirable advances in neuroprognostication would provide a high degree of certainty that (1) when the prognosis is good, continued treatment will indeed yield a good outcome and (2) when the prognosis is poor, continued treatment would not yield a good outcome.

Note the difficulty in testing a new prognostic method for satisfaction of that second desideratum. Conducting an appropriate experiment carries an immense moral risk. At worst, it is unacceptably inhumane. If there is a credible prediction of a poor outcome, then the only straightforward way to validate this prediction is to continue treatment and observe whether this indeed yields a poor outcome.^15^ However, validating the prediction would entail bringing about exactly the sort of outcome, such as an unacceptable quality of life for the patient, that decision-makers aim to avoid. Confirming the prediction by continuing treatment when it has been credibly predicted that this will yield a poor outcome is thus not beneficent to the patient and is arguably maleficent. Hence, core principles of biomedical ethics^16^ favour discontinuation of therapeutic and life-sustaining treatment. In short, ethical treatment of patients demands the SFP, even though the SFP precludes the very observations that would be required to test the theories and technology on which the prediction was based.

Thus, we have an additional dilemma, a choice between moral value in clinical practice and epistemic value in research: choosing either to prioritise the interests of the individual patient or to advance research to benefit future patients. Note that this dilemma, which is due to the innovation practice being at the intersection of research and care, is entirely distinct from the well-recognised clinical dilemma described above. This collision of values occurs precisely because the only relevant research subjects are necessarily also patients. It is a paradigm case of liminal innovation practices, in which the research-practice distinction cannot be upheld,^17^ and competing aims come into conflict.

In sum, observing which positive test results turned out correct and which turned out incorrect is required for assessing the quality of prognostic tests. SFPs obscure precisely this distinction. So, when SFPs occur, any feedback we seem to get about the success of our tests is not genuinely informative. Hence, SFPs prevent us from making valid assertions about the quality of our tests. Researchers should thus be hesitant to assert a precise false positive rate or precise specificity value of tests of poor outcome, when any cases of WLST cause SFPs.^7^ Thus, for the sake of research, avoiding SFPs is of vital importance. However, as just explained, preserving the interests of patients often strongly favours enacting the self-fulfilling prognosis.

## Three ways that the SFP distorts prognostic research

We have shown that, in research, when SFPs occur, we cannot tell the difference between false and true positives. However, we know from cases where life-sustaining treatment was not withdrawn, that false positives do occur, even with the most reliable tests.^21^ ^8^ If there are indeed false positives, they remain hidden after WLST due to the epistemic challenge of SFPs. We now describe three ways in which these hidden errors can persist through various iterations of prognostic innovation.

First, past SFPs hide past false positive results occurring in established testing protocols, thus influencing clinical decision making in the contexts in which novel tests are being evaluated.^17^ Cases of this type proceed along the lines of this pattern: A new prognostic test is being evaluated. In the case of postanoxic coma, this may be a combination of continuous electroencephalogram (cEEG) monitoring with machine learning for data analysis.^22^ Unknown to the treating clinician, who is kept blinded from the new test, the new test outputs a prognosis for a particular patient. Yet care for this patient proceeds not according to the deliverances of this novel test, but according to a series of established testing methods, perhaps including a somatosensory evoked potentials (SSEP) test. If one of the established test results is positive, even if it is a *false* positive, the ensuing poor prognosis justifies (perhaps even demands) WLST. This constitutes a poor outcome, and this is the very outcome recorded and compared with the prediction derived from the novel test. Hence, any bias towards false positives in the established test is present in the evaluation of the novel test, and is thus propagated to the novel test as it is calibrated.

This problem would be averted only if the established tests were perfectly accurate, or at least perfect in their specificity, never issuing false positives. But this will likely not be the case. One primary motivation for the development of a new test is to improve on the accuracy of the established tests. SSEP tests, for instance, have an estimated false positive rate of 7.7%.^23^ ^9^ Hence, the self-fulfilment of the prognosis obscures the fact that bias towards false positives in the earlier generation of tests informs clinical practice in ways that distort the evaluation of novel tests. To avoid letting new techniques inherit the undiscovered false positives of established techniques, estimates of extant false positive rates (if only statistically determined^2 23^) must be considered when evaluating the new test.

The second distorting influence of SFPs is apparent when research and practice overlap, either in physical space, with the instrumentation involved, or with the personnel involved. In practice, it is difficult to prevent such overlaps. In fact, according to one recent review, only 9 out of 73 neuroprognostic studies reported blinding treating physicians from research results.^12^ A case of research-practice overlap may unfold as follows: The clinician in charge of prognosis and decision making is not blinded to the instrumentation being tested. So, even if the established prognostic test is SSEP, an electroencephalogram (EEG) machine that is part of a novel test may be present in the room with the patient and the physician.^22^ This invites a natural form of confirmation bias.^24^ Any hypothesis entertained about the evidential value of indicators in the new instrumentation affects not just the perceptions of the researchers but also the decision making of the clinician (who may well be one of the researchers). So, an initial hypothesis about the deliverances of a new test affects treatment decisions, thus affecting the patient outcomes. In particular, if the hypothesis suggests a poor prognosis and this influences treatment decisions accordingly, the prognosis becomes self-fulfilling thereby spuriously confirming the hypothesis. So, even if the suspected positive was a false positive, this cannot be noticed. The evidence available shows the positive test result as a true positive. The consequence is that any deficit of specificity in the new test is made invisible. Quite appropriately, then, we already see criticism of prognostication studies for lacking the sort of blinding that we have come to expect in other sorts of clinical trials.^2 10 11^

The seemingly obvious solution would be to blind the treating physicians from all instruments and data that are part of the research. But this is precisely where we must face the fact that the division between medical research and practice is not sharp.^17^ ^25^ Although blinding the treating physicians would help ensure the integrity of the research, this may not always be consistent with providing the best possible care to the patient. For instance, the data from cEEG-monitoring can also be used to detect epileptic activity, which may subsequently be treated with antiepileptic drugs. Other times, there just may be no better prognostic information available than what comes from the experimental test, and the treating physician may have no choice but to explicitly request the data they were blinded from. Thus, even if the physician in charge of prognosis is typically blinded from the administration of the novel test being tested, patient care may favour using some of the instruments involved in the novel test. Blinding treating physicians should still be encouraged, whenever it is consistent with patient care. However, as soon as research data informs treatment of a patient, it is responsible research practice to take that patient out of the data set or at least report the non-blinded element.^26^ Otherwise, unrecognised false positives due to confirmation bias will pollute the data regarding the accuracy of new tests and hence misinform prognosis for future patients.

The third kind of distortion is the forward-looking version of the first kind. As a new test emerges from the realm of research and is put into practice, a series of new instances of prognosis are based on this test. Assuming the new test is not perfect in its specificity, there will still be false positives, which will then be hidden, as described before, by self-fulfilling prognoses of poor outcome. Just as SFPs based on an earlier prognostic method had the potential to distort research on a novel test, these new SFPs become a new source of distortion when there is an attempt to refine or automate the novel test. To see this, consider the novel cEEG-based test. Each time the new test is used, we have EEG readings paired with a record of the eventual outcome for the patient in question. As the test is run again and again with new patients, we have the accumulation of a large, valuable data set. The accumulating data has exactly the right structure to serve as the training and testing datasets needed to refine prognostic algorithms through machine learning.^22^ ^18^ The problem is that, due to self-fulfilment of prognoses, the eventual outcomes will be poor whenever a poor outcome was predicted. As a result, new data for refining the test will contain uncorrected false positives. Hence, what is ‘learnt’ is precisely the pattern of prognostic judgements already encoded in the predictive methods intended to be refined. Thus, we have a potential for amplified bias through a feedback loop.^27^ At best, any bias towards false positives remains uncorrected and hinders improvement of the test’s specificity. At worst, bias is amplified as the test becomes more and more biased towards a positive result.

The obvious solution is to not train new models on data influenced by the use of current tests, but, just like with the exclusion of patients in the previous problem, this exclusion of potential training data may leave insufficient amounts of suitable data available. This deficit can, to an extent, be resolved by conducting collaborative research in countries where WLST is uncommon or prohibited. Specifically, researchers need clean data on patient outcomes (from places where those outcomes are not affected by early withdrawal) as well as prognostic readings about those patients. We are optimistic about this possibility and see the results of research in countries where life-sustaining treatment is less often withdrawn^28^ strengthening and validating research in countries where it is commonly withdrawn earlier.^19^ That said, in contexts where WLST is uncommon, varying standards makes comparisons of results difficult. Other factors influencing outcomes like heightened pressure on limited resources, suboptimal intensive care, soft codes (eg, softer and shorter cardiopulmonary resuscitation), etc. must be taken into consideration.

In sum, undiscovered false positives due to SFPs caused by WLST impede improvement of new prognostic tests in three ways, each of which incorporates bias into research. The result, in each case, is that false positive rates of novel tests will likely appear lower than they actually are, and specificity will likely appear higher than it actually is. The earlier and the more often life-sustaining treatment is withdrawn, the worse this problem is.

## Exacerbating the asymmetry in our precautionary biases?

Before concluding, we wish to briefly point out a particular risk that a bias towards SFPs in life-and-death cases of neuroprognostication may be naturally self-reinforcing. The risk is that, if the epistemic issues we have been describing are not addressed, the moral concerns that have prompted ethicists and clinicians to worry about SFPs will tend to worsen rather than alleviate.

In neuroprognostication for patients in coma, both false negatives and false positives are tragic. However, as we have seen, only false negatives are evident. In case of a false negative or any mistakenly optimistic prognosis, not only is the erroneous prediction apparent, it typically results in a quality of life the patient and the patient’s loved ones never would have found acceptable. Hence, the cases are often experienced as distressing and sorrowful, providing strong motivation to avoid such cases in the future, to whatever extent possible. The evident error delivers powerful feedback to improve accuracy for negative test results, but no such feedback exists for positive test results that yield SFPs. As already emphasised, in cases of false positives that result in SFPs, there is no error signal, that is, no readily apparent evidence that anything was amiss with the prediction (table 1). Clinicians are regularly confronted with these highly undesirable consequences of false negatives but never with those of the false positives that yield SFPs. Understandably, this evidential asymmetry may incline clinicians and researchers to increasingly lean towards predictions of poor outcome to correct for false negatives. But there is definitely no counterpart pressure^10^ to correct for false positives and lean towards predictions of good outcome.

This evidential asymmetry we observed in practice comes on top of an already existing asymmetry between the ways we evaluate harms and benefits. When faced with a choice between a risk of great harm or a loss of comparable or even greater benefit, precaution already creates a bias that favours avoidance of harm.^30^ In treatment of an adult patient in coma, where a choice has to be made between risking a terrible outcome, and risking the loss of a valuable one, this precautionary bias can be palpable.^11^ With such cases, it often makes sense for clinicians and family members to favour avoiding the harm of subjecting a loved one to a severe disorder of consciousness or other unacceptable quality of life.

This precautionary bias further favours the avoidance of false negatives, which compounds the evidential asymmetry between false positives and false negatives caused by SFPs. This means that, especially when predictions are employed more often or earlier, more and more patients are likely to not receive treatment that might have provided them a prolongation of a valuable life. Hence, more patients could meet an unnecessary, untimely demise. With that, we run head-on into the ethical consequences of the epistemic difficulties we have been unravelling. The moral concern that draws attention to SFPs in neuroprognostication is thus prone to be exacerbated by the consequences of SFPs’ epistemic problems.

## Conclusion: facing the epistemic importance of SFPs

In this article, we have shown how SFPs distort research and innovation of new techniques for neuroprognostication. While practitioners in clinical practice have long been concerned about transformative SFPs that change the outcome of the patient, we have argued that responsible innovation in neuroprognostication requires more attention to *all* SFPs, including operative ones that do not change the outcome of the patient. Only with this wider attention to SFPs is it possible to address the epistemic problems they raise.

The core epistemic problem of SFPs in research is that the existence of self-fulfilment, whether transformative or merely operative, impedes informative feedback regarding the accuracy of positive test results. Not being able to distinguish, in practice, between transformative and operative SFPs means we cannot distinguish between true positives and false positives. This both prevents detecting false positives and impedes improvement of the specificity of our tests.

Responsible innovation requires a more careful approach with respect to each of the three ways in which SFPs distort research. The following three guidelines are relevant not only to the researchers themselves, but also to the peer reviewers who should integrate this guidance into their quality assurance process.

1. To avoid undiscovered false positives from established technology propagating to new technology, it is imperative to take into account the old test’s unbiased estimates of false positives, adjusted for WLST, when assessing the new test.
2. When possible, treating medical staff should be completely blinded from neuroprognostic studies. Whenever research data are explicitly requested to inform treatment of a patient, responsible research practice requires taking that patient out of the data set.
3. Ideally, new models should not be trained on data influenced by the use of current tests. In research where models have been trained on tainted data, this problem should be clearly flagged and strategies for mitigation should be discussed.

In cases where adherence to these guidelines poses a challenge to collecting sufficient research data, alternatives ought then to be explored. We recommend considering collaborations with researchers in other countries where there are different practices regarding WLST, keeping in mind other limitations these contexts may have.

In general, it is worth reemphasising the upshot of the core challenge posed by SFPs: When predictions bring about their own fulfilment, researchers can never rely on, as genuinely informative, any feedback they receive about the accuracy of those predictions.

Learning is a fundamental part of research and innovation. If we have an innovation process, or research method, that limits how much we can learn from our successes and failures, that research fails to do what research is supposed to do, namely produce accurate learning. Responsible learning requires attention not just to the mistakes that are most apparent to us. It also demands that we heed the mistakes that are hidden from us, as with prognoses that are self-fulfilling.

## Footnotes

The classic conception of self-fulfilling prophecy is from sociologist Merton.^1^ Our definition departs from Merton’s in ways that make the notion of SFP suitable for analysing its epistemic implications. In particular, we allow that a prediction that brings about an outcome that still would have been otherwise realised counts as an SFP. Our definition also departs from Dominic Wilkinson’s analysis of self-fulfilling prophecies in intensive care, according to which these prognoses are best understood as ‘self-reinforcing’ in that they “increase the probability of an outcome occurring that had some chance of occurring otherwise.”^2^ According to our conception, a particular prognosis is an SFP *only if* the outcome predicted is *actually* realised, and this realisation is due to the prediction.

Dichotomising neurological outcomes after coma based on Cerebral Performance Categories into good and poor is standard practice in neuroprognostic research, though the delineation between the two has shifted over time.^6^

We will not engage in the discussion of whether or when WLST in case of poor prognosis is morally justified. We start from the assumption that in some cases it can be highly reasonable to withdraw treatment on the basis of a poor prognosis^4 5^—a starting point that also informs legislation on WLST in an increasing number of countries.

Empirical study of neuroprognostic innovation practices, conducted by the first author, as well as clinical experience of the fourth author, also confirms the prevalence of WLST in response to poor outcome prediction.

Accordingly, false positives for brain death are even more difficult to catch. That is because when brain death has been diagnosed this (almost) always results in WLST, and so there are (almost) never opportunities to catch incorrect diagnoses. In other words, the diagnosis of brain death is inevitably an SFP, unlike prognoses of poor outcome for coma patients which are commonly, but not always, SFPs.

Not all negative test results entail a prognosis of good outcome. The somatosensory evoked potential (SSEP) test, for instance, only reliably predicts poor outcomes. A negative result, as such, does not predict a good outcome but can still motivate continuation of treatment.

Some studies report 0% false positive rates and 100% specificity for poor outcome predictions.^18–20^ It is wise for diligent reviewers of such studies to question such results in light of the pitfalls we have described.

As Maciel and colleagues explain, citing twelve different studies, ‘Accumulating reports of survival with good neurologic recovery despite low likelihood based on one or multiple prognostic factors challenge current neuroprognostication practices.’ They continue, ‘even findings considered infallible in predicting poor outcome…have lost their immaculate prognostic ability, although they continue to have very low false positive rates.’^21^

While most studies report much lower false positive rates for SSEP, these studies are subject to precisely the kind of bias due to SFP we discuss. The study done by Amorim and colleagues (2018)^23^ aimed to develop an unbiased estimate by adjusting for WLST.

Sometimes life-sustaining treatment is continued despite a poor prognosis.^29^ A recovering patient then typically affects treating physicians in the opposite direction, making them (at least temporarily) significantly more careful about withdrawal.

In contrast, one can imagine that—with newborns in coma—the potential loss of an expected child is a harm that may flip the precautionary bias.
